# IFNγ prolongs survival in experimental *Escherichia coli *pyelonephritis: implications for favorable phagocytosis

**DOI:** 10.1186/cc9670

**Published:** 2011-03-11

**Authors:** M Katsaris, T Adamis, M Georgitsi, A Pistiki, M Chrisofos, E Giamarellos-Bourboulis

**Affiliations:** 1 University of Athens, Medical School, Athens, Greece; 2 Attikon University Hospital, Athens, Greece

## Introduction

IFNγ is a promising immunomodulator in sepsis because it is thought it may reverse immunoparalysis and improve phagocytosis. Its effect was investigated in experimental pyelonephritis and sepsis.

## Methods

Experimental pyelonephritis by *Escherichia coli *was induced in 18 rabbits after ligation of the right pelvo-ureteral junction and infusion of one 1 × 10^7 ^log-phase cfu/ml inoculum above the ligation. Animals were assigned into 10 controls (group A) and into eight administered intravenously 0.1 μg/kg IFNγ 30 minutes after bacterial challenge (group B). Blood was sampled at serial time intervals; quantitative cultures were done; apoptosis of lymphocytes and of monocytes was assessed by flow cytometry; malondialdehyde (MDA) was estimated by the thiobarbiturate assay and passage through an HPLC system. After death, quantitative tissue cultures were done.

## Results

Median survival of group A was 3 days and of group B was 18 days (log-rank: 4.858, *P = *0.028). Mean log_10 _of bacteria in blood for groups A and B at 2 hours was 1.59 and 1.21 (*P = *NS); at 4 hours 1.61 and 1.97 (*P = *NS); at 24 hours 1.28 and 1.02; and at 48 hours 1.29 and 1.00 (*P = *NS). Respective rates of apoptosis of lymphocytes at 2 hours were 17.1 and 22.2% (*P = *NS); at 4 hours 17.9 and 24.0% (*P = *NS); at 24 hours 18.3 and 21.9% (*P = *NS); and at 48 hours 20.5 and 22.8% (*P = *NS). Respective rates of apoptosis of monocytes at 2 hours were 32.8 and 36.0% (*P = *NS); at 4 hours 42.8 and 39.3% (*P = *NS); at 24 hours 54.5 and 62.1% (*P = *NS); and at 48 hours 52.5 and 64.3% (*P = *0.042). Respective median serum MDA of groups A and B were 1.05 and 2.06 μmol/ml at baseline (*P = *NS); 0.93 and 2.54 μmol/ml at 2 hours (p: 0.028); 2.30 and 1.02 μmol/ml at 4 hours (*P = *NS); 1.47 and 2.05 μmol/ml at 24 hours (*P = *NS); and 1.71 and 1.85 μmol/ml at 48 hours (*P = *NS). Mean log_10 _of bacterial growth in the liver of group A and of group B on sacrifice was 3.47 and 1.32, respectively (*P = *0.043); and in the right kidney was 5.78 and 1.94, respectively (*P = *0.004).

## Conclusions

IFNγ prolongs survival when administered after induction of experimental pyelonephritis by *E. coli*. Its effect is mediated through: enhanced phagocytosis as evidenced by increase of oxidant stress and decrease of tissue bacterial load; and modulation of inflammation as evidenced by increase of apoptosis of monocytes.

